# Fatal Effects of Eating Cherries, and Copious Bleeding

**Published:** 1811-01

**Authors:** J. E. Harrison

**Affiliations:** Thornbury, Gloucestershire


*To the Editors of the Medical and Physical Journal.*

Gentlemen,

My son, who studied physic at Philadelphia in America, relates the following case, which occurred whilst he was attending the hospital in that city.

"The porter belonging to the Hospital (Philadelphia) having eaten excessively of cherries, which abound there, was seized with violent vomiting and convulsions. It was Dr. Rush's month of attending. The House Surgeon bled the man freely without relief; in the evening he was bled again, copiously, in both feet: the next day the arteries in both wrists were opened ad deliquium, without relief, the convulsions continuing. The day after, both arteries in the ancles were opened ad deliquium. The following morning, being the fourth day from the attack, he expired in convulsions."

My son makes the following observations.—If the patient, as he vomited up the cherries, had taken plentifully of warm water or camomile tea, to have emptied his stomach, as nature dictated an emetic, and then a purging mixture, or enema, it is my opinion that his life might have been saved.

A gentleman in very extensive practice, in a hurry, opened an abscess in the throat with a lancet which he had used for inoculation. The child a few days afterwards was seized with a putrid fever. I saw the child; it was about two years old. The part that was opened had hundreds of the confluent small-pox; no other part of the body, excepting the place where the abscess was opened, was affected. The child expired covered with purple petechiæ in a very few days. The gentleman affirms, that the lancet had been well scalded in boiling hot water before he used it.

I am yours, &c.

*Thornbury, Gloucestershire,* J. E. HARRISON, M. D.

*Nov*. 26, 1810.

Note. Dr. Harrison requests us to correct an omission in his Case of Cancer, in the Journal for October 1810, p. 276, line 6, where instead of advising the patient to trust, it should have been written "advising her to trust in God." And in the case of Fluor Albus, p. 279, for discharge remaining, read *No* discharge remaining.

